# No Longer Aging in Place: Housing Decisions After 100

**DOI:** 10.1093/geroni/igaa057.169

**Published:** 2020-12-16

**Authors:** Susan Bowers

**Affiliations:** Northern Illinois University, sycamore, Illinois, United States

## Abstract

The number of aging individuals is growing and, along with it, a subset of the oldest-old (those over 85 years), including centenarians. Although researchers have begun identifying issues and needs related to this population (Dunkle & Jeon, 2016), still little is known about decision-making processes as they relate to housing. In rural areas, in specific, centenarians are limited by few residential choices and lack of geographic mobility. In this study, decision-making processes are examined, with an emphasis on interactions between aging individuals and their rural family caregivers. In addition, since family caregivers typically experience a pattern of burnout over time (Yilmaz, Turan & Gundogar, 2009; Yikikan, Aypak, & Görpelioğlu, 2015), a second focus of the study is caregiver stress. Data for the study are drawn from semi-structured interviews with a sample of family caregivers in the Midwest. All caregivers had a 100-plus family member recently placed, or in process of placement, at a residential long-term care facility. To meet criteria, all facilities were in towns of 4000 individuals or less. Data consisted of qualitative interviews with the primary family contact (female in all cases), and were analyzed according to Strauss & Corbin (1990). Decision-making themes centered on health and family pressure. Stress themes centered primarily around work. Data are discussed in terms of family strengths, health and wellness, and the need for continued programming for family caregivers, particularly in rural areas.

